# Prolonged haematologic toxicity in CAR‐T‐cell therapy: A review

**DOI:** 10.1111/jcmm.17930

**Published:** 2023-09-13

**Authors:** Qi Liu, Tonglin Hu, Hangchao Li, Yingying Shen, Dijiong Wu, Baodong Ye

**Affiliations:** ^1^ Department of Hematology The First Affiliated Hospital of Zhejiang Chinese Medical University (Zhejiang Provincial Hospital of Chinese Medicine) Hangzhou China; ^2^ The First School of Clinical Medicine Zhejiang Chinese Medical University Hangzhou China

**Keywords:** CAR‐T‐cell therapy, management, mechanism, prolonged haematologic toxicity

## Abstract

Chimeric antigen receptor‐T‐cell (CAR‐T‐cell) therapy is a novel immunotherapy with encouraging results for treatment of relapsed/refractory haematologic malignancies. With increasing use, our understanding of immune‐mediated side effects such as cytokine release syndrome and neurotoxicity has improved; nevertheless, prolonged haematologic toxicity (PHT), with a high incidence rate, remains underrecognized. Owing to heterogeneity in populations, the CAR‐T cells used and diseases treated as well as differences in the definition of PHT, its rate, risk factors and management vary across studies. In this review, we provide a narrative of PHT occurring in patients following CAR‐T‐cell therapy; evidence of PHT treatment strategies is also presented, with the aim of contributing to systematic understanding of PHT.

## 1 INTRODUCTION

Chimeric antigen receptor‐T‐cell (CAR‐T‐cell) therapy is a novel immunologic interventional approach approved by the Food & Drug Administration for relapsed or refractory haematologic malignancies. The key components of CAR‐T‐cell products are extracellular domains for tumour antigen recognition, such as CD19, CD20, CD22 and B‐cell maturation antigen, and intracellular costimulatory domains, such as 4‐1BB and CD28, which are crucial for T‐cell activation. These modified CAR‐T cells are infused into the patient's body following the lymphodepleting conditional regimen, with sustained lethality to the tumour through the patient's immune system.

Worldwide clinical trials have demonstrated the efficacy of CAR‐T‐cell therapy in both relapsed/refractory (R/R) haematologic malignancies and solid tumours.^1^, ^2^, ^3^, ^4^ However, CAR‐T‐cell therapy is also linked to a series of toxicities, such as cytokine release syndrome (CRS), immune effector cell‐associated neurotoxicity syndrome and B‐cell aplasia, which have drawn much attention and are largely reported to be attributed to unique adverse events different from those associated with standard chemotherapy.^5^, ^6^, ^7^ Previous studies have reported a high incidence of haematologic toxicity (HT), including neutropenia, anaemia and thrombocytopenia, after CAR‐T‐cell therapy, with biphasic characteristics.^5^, ^8^, ^9^ Cytopenia due to CAR‐T‐cell therapy is classified according to occurrence and duration into early and prolonged haematologic toxicity (PHT).^5^ Although the definition of PHT varies in research, the majority of studies define PHT as unresolved grade 3/4 cytopenia (according to the National Cancer Institute's Common Terminology Criteria for Adverse Events) at Day 28 after CAR‐T‐cell therapy. Importantly, early HT is considered to be common and expected due to the myelotoxic effect of lymphodepleting chemotherapy prior to CAR‐T‐cell infusion, with an incidence greater than 90%.^5^ In the long‐term follow‐up of the ZUMA‐1 study, 17% of patients developed PHT (grade 3 or worse cytopenia) after 3 months.^8^ Moreover, nearly 11% of patients did not achieve haematopoietic recovery at 12 months after CAR‐T‐cell infusion.^10^

PHT actually exceeds the expectations of chemotherapy and continues for a long time after resolution of CRS. Of note, PHT and delayed haematopoietic recovery may result in an increased frequencies of infection, haemorrhagic events, blood product transfusions and prolonged hospitalization, which usually contribute to decreased quality of life, treatment‐related morbidity and mortality.^11^, ^12^ However, the mechanism of PHT is not yet clear and needs to be investigated. Here, we systematically summarize the current literature regarding PHT after CAR‐T‐cell therapy and present recommendations for its prevention and treatment.

## 2 INCIDENCE OF PROLONGED HAEMATOLOGIC TOXICITY AFTER CAR‐T‐CELL THERAPY

A narrative review was performed using the PubMed database (censored April 2023) with the following search terms: CAR‐T, chimeric antigen receptor, HT, neutropenia, anaemia, thrombocytopenia and cytopenia.

We retrieved 34 studies that specifically investigate rates of PHT in patients undergoing CAR‐T therapy, though the definitions of prolongation and severity of cytopenia varied (Table S1). Overall, all studies defined PHT according to the time of occurrence, except for Kato's study, which was based on the duration of PHT.^13^ Fried et al. defined PHT as any grade of neutropenia, anaemia or thrombocytopenia on Day 21 after CAR‐T‐cell infusion and found that more than 90% of patients experienced prolonged cytopenia^5^; the incidence of PHT decreased to 78.9% on Day 28 in the Wang cohort^14^ and to 66.7% in the Kitamura study.^15^ The standard for the severity of PHT in most studies is unresolved grade 3/4 cytopenia. Under this standard of severity, eight studies analysed the cumulative incidence on Day 28,^16^, ^17^, ^18^, ^19^, ^20^, ^21^, ^22^, ^23^ two studies on Day 29,^24^, ^25^ 10 studies on Day 30,^26^, ^27^, ^28^, ^29^, ^30^, ^31^, ^32^, ^33^, ^34^, ^35^ one study on Day 35,^36^ one study on Day 42^37^ and three studies on Day 90^8^, ^38^, ^39^ after CAR‐T‐cell infusion. In general, the rate of grade 3 or worse cytopenia ranged from 20% to 77.8% on Day 28 to Day 35 after CAR‐T‐cell infusion,^18^, ^30^ and differences in the incidence of PHT were ascribed to heterogeneity in the populations, CAR‐T cells used and diseases treated. As noted, the incidence of PHT declined to approximately 20% after 90 days.^8^

Some studies also reported specific rates of blood lineage reduction after CAR‐T‐cell therapy. Rates of grade 3 or 4 neutropenia occurring on Day 30 after CAR‐T‐cell therapy were 38.71%, 29%, 25% and 32%, compared to 22.58%, 16%, 7% and 7% for corresponding anaemia and 59.14%, 42%, 18% and 10% for thrombocytopenia, respectively.^26^, ^28^, ^31^, ^32^ Furthermore, it has been reported that the median time to recovery to grade 2 or lower for prolonged thrombocytopenia is 2.1 (range 1.2–13.8) months, longer than prolonged neutropenia recovery, at 1.9 (range 1.2–5.6) months.^34^ The same results were obtained in Abramson's study, which showed that 37% (100/269) of patients experienced PHT on Day 29; among them, 84% (36/43), 82% (9/11) and 62% (36/58) of cases recovered to grade 2 or lower neutropenia, anaemia and thrombocytopenia by Day 90, respectively; the rate of haematopoietic recovery increased to 93% (40/43), 91% (10/11) and 74% (43/58) by Day 180, respectively.^25^ It is worth noting that neutropenia is more likely to occur after Day 30 after infusion, followed by thrombocytopenia and anaemia; however, thrombocytopenia is maintained for a longer time and is simultaneously more difficult to reverse. Overall, the majority of cases with grade 3 or worse prolonged cytopenia recovered to grade 2 or better by Day 90 after CAR‐T‐cell infusion.^22^, ^36^ According to the literature, PHT is common but reversible.

## 3 RISK FACTORS FOR PROLONGED HAEMATOLOGIC TOXICITY

Seven of the previously mentioned studies investigated factors associated with increased risk of PHT among their cohorts. Fried et al. found that prior haematopoietic stem cell transplant (HSCT) and higher CRS grade correlated positively with late cytopenia on Day 21; synchronous changes in C‐X‐C motif ligand 12 (CXCL12) and late neutropenia were observed post CAR‐T therapy.^5^ Based on univariate analysis, Eastern Cooperative Oncology Group performance status 1, >3 prior therapies and low absolute lymphocyte count were related to Day 30 grade 3–4 cytopenia in the Strati cohort.^28^ Juluri et al. used the debiased least absolute shrinkage and selection operator for high‐dimensional linear regression modelling and identified that CRS severity was predictive of Day 28 platelet count and prelymphodepleting platelet count and that the transforming growth factor beta‐1 level was a protective factor for both Day 28 absolute neutrophil count (ANC) and platelet count.^40^ Li et al. used univariate and multivariate logistic regression models of baseline characteristics, serum cytokine levels and CAR‐T‐cell therapy‐associated factors and identified interferon‐gamma (IFN‐γ) and severe HT after lymphodepleting chemotherapy as independent risk factors for PHT on Day 28 after infusion.^41^ Similarly, Wang et al. found that cytokine profiles were independent risk factors for PHT; specifically, multivariate analysis determined high maximum interleukin‐10 (IL‐10) and IL‐17A levels as independent risk factors for prolonged neutropenia, high maximum IL‐6 for prolonged anaemia and high IL‐2 baseline levels as protective factors for prolonged thrombocytopenia.^26^ Moreover, CAR‐HEMATOTOX, a model for CAR‐T cells related to HT in patients with lymphoma, comprises markers of haematopoietic reserve (baseline platelet count, haemoglobin and ANC) and baseline inflammation (C‐reactive protein [CRP] and ferritin), which can predict a longer duration of neutropenia.^9^ Similarly, Nagle et al. confirmed the association between peak ferritin and CRP levels in combination with CRS and development of PHT by univariate analysis.^27^ Factors related to PHT are shown in Table 1.

**TABLE 1:** Factors related to prolonged haematologic toxicity.

Risk factors	Protective factors
Prior HSCT	Higher sdf‐1 and TGF‐β1
>3 prior therapies	Baseline haematopoietic function (baseline PLT, HB, ANC and ALC)
ECOG performance status 1	Higher baseline IL‐2
Severe haematologic toxicity after lymphodepleting chemotherapy	
Higher CRS grade	
Higher baseline ferritin and CRP	
Higher maximum IFN‐γ, IL‐6, IL‐10 and IL‐17A	

Abbreviations: ALC, absolute lymphocyte count; ANC, absolute neutrophil count; CRP, C‐reactive protein; CRS, cytokine release syndrome; ECOG, Eastern Cooperative Oncology Group; HB, haemoglobin; HSCT, haematopoietic stem cell transplantation; IFN‐γ, interferon γ; IL, interleukin; PLT, platelet; sdf‐1, stromal cell‐derived factor 1; TGF‐β, transforming growth factor‐b1.

## 4 MECHANISMS OF PROLONGED HAEMATOLOGIC TOXICITY

To date, several studies have attempted to identify factors contributing to PHT, such as prechemotherapy regimens, severe CRS and baseline haematopoietic function.^5^, ^42^ However, the underlying mechanisms of PHT after CAR‐T‐cell therapy remain unclear.

### 4.1 Impaired bone marrow microenvironment and haematopoietic stem cells

Kitamura et al. were the first to study changes between the bone marrow (BM) niche before and after CAR‐T‐cell infusion and its relationship with PHT, reporting that PHT patients tended to have a lower area proportion of CD271^+^ cells, which are proposed as numbers of HSPC niches, lower levels of CXCL12 and SCF in BM before CAR‐T‐cell infusion, and increased inflammation‐related cytokine levels of IL‐6 and monocyte chemoattractant protein‐1 in BM.^15^ In addition, higher levels of vascular endothelial growth factor (VEGF) and macrophage‐derived chemokines have been detected in patients with complete haematopoietic recovery,^10^ revealing the mechanism of PHT from the BM microenvironment perspective. Furthermore, patients with PHT showed hypoplastic, as confirmed by BM biopsies in the Nagle cohort,^27^ resembling BM manifestations of acquired aplastic anaemia (AA). Immune‐mediated disruption of haematopoietic stem cells (HSCs) is a partial mechanism of acquired AA. Strati et al. observed delayed reconstitution of CD4^+^ T cells in three of nine (33%) patients with PHT at 1 year and two of seven (29%) at 2 years; persistent grade 3–4 cytopenia occurred in 27% (4/15) at 1 year and 11% (1/9) at 2 years, indicating that immune reconstitution was delayed compared with haematopoietic recovery.^28^ All this evidence demonstrates that imbalanced immune homeostasis and BM failure contribute to development of PHT after CAR‐T‐cell therapy.

### 4.2 Clonal haematopoiesis and secondary myelodysplastic syndrome

The occurrence of clonal haematopoiesis or myelodysplastic syndrome (MDS) may contribute to PHT development, especially in patients who receive multiple cytotoxic therapies prior to CAR‐T‐cell infusion.^8^, ^43^ Nahas et al. reported that two of eight patients with severe neutropenia at 42 days after CAR‐T‐cell infusion displayed definitive evidence of MDS characteristics (one with 7q‐ and excess blasts and another with 20q‐ and multilineage dysplasia).^44^ In the study by Strati et al. three of 15 patients with PHT were diagnosed with MDS, with a median time to onset of 13.5 (range 4–26) months.^28^ Kochenderfer et al. reported one of seven cases of complete remission of DLBCL with new cytopenia at 39 months after anti‐CD19 CAR‐T‐cell therapy diagnosed as MDS.^45^ Unfortunately, information about clonal haematopoiesis‐related MDS prior to CAR‐T‐cell infusion is lacking for these cohorts. However, Cordeiro et al. found that two of four patients with a subsequent diagnosis of MDS had cytogenetic abnormalities before receiving CAR‐T‐cell therapy (one harboured *t*[10;13], and the other del5q).^43^ What drives the occurrence of MDS? It has been reported that secondary MDS following ASCT is associated with previous exposure to topoisomerase II inhibitors and alkylating agents.^46^ Many patients have received fludarabine and cyclophosphamide as lymphodepleting chemotherapy before CAR‐T‐cell infusion, which may increase the risk of developing MDS. In addition, the possibility that CAR‐T‐cell treatment itself may lead to the occurrence of MDS cannot be excluded. Recently, a case report described a patient diagnosed with acute myeloid leukaemia after CAR‐T‐cell therapy. *DNMT3A* and *PPM1D* mutations already existed before treatment, but with newly acquired *RUNX1* mutation.^47^ Accorsi Buttini et al. also reported that one patient with R/R DLBCL based on sustained *CSF3R* and *CEBPA* mutations developed secondary MDS and acquired a new *RUNX1* mutation after CAR‐T‐cell therapy.^48^ These two cases suggest that CAR‐T‐cell treatment is similar to a double hit to promote development of MDS for those who already have clonal haematopoiesis prior to CAR‐T‐cell therapy. Conversely, Zhao et al. reported an incidence of therapy‐related myeloid neoplasms after CAR‐T‐cell therapy of 3.2%, comparable to that in patients who received chemotherapy or HSCT; hence, they deemed that CAR‐T‐cell infusion did not significantly increase the risk of therapy‐related myeloid neoplasms according to the current data for incidence.^49^ Moreover, there was no significant difference in the incidence of MDS between patients with and without PHT from ZUMA‐1 and ZUMA‐9 in the Strati cohort (3/15 [20%] vs. 1/16 [6%], *p* = 0.33).^28^ Briefly, whether CAR T cells themselves participate in the secondary MDS is controversial. Further research is needed for better comprehension of the pathophysiology of MDS post CAR‐T‐cell therapy.

### 4.3 Sustained inflammation‐mediated impairment

Another possible reason for the emergence of PHT may be inflammation due to CAR‐T‐cell therapy. Several studies have linked PHT to high‐grade CRS, inflammation markers (i.e. ferritin and CRP), and tocilizumab or steroid treatment,^5^, ^27^, ^44^ which all indicate that patients with a greater degree of inflammation are more likely to experience PHT. Notably, some studies have discovered an association between cytokines and prolonged cytopenia.^15^, ^26^, ^40^, ^41^ These cytokines not only have proinflammatory and immunomodulatory properties but also different functions in haematopoiesis. IL‐2, a lymphocyte‐specific growth factor, plays a critical role in maintaining erythropoiesis by modulating Treg activity in BM,^50^, ^51^ which may explain the protective effect of IL‐2 against PHT. IL‐6 is a robust proinflammatory cytokine that participates in immune and inflammatory responses.^52^ The latest research demonstrates the prominent role of IL‐6 in haematopoietic ageing. IL‐6 is upregulated in the aged microenvironment and is associated with decreased functionality of erythroid progenitor populations, which can be alleviated by IL‐6 inhibition,^53^ and IL‐6 can promote emergency granulopoiesis and differentiation of the monocyte–macrophage lineage in response to inflammatory stimulation.^54^ IL‐10 is an anti‐inflammatory cytokine and can trigger emergency myelopoiesis.^55^ Recent research discovered that the level of IL‐10 was increased in the spleen, serum and BM in a benzene‐induced HT mouse model, implying a damaging effect of IL‐10 on haematopoiesis.^56^ IFN‐γ can active quiescent HSCs in response to chronic infection and thus may exhaust them, resembling the manifestation of AA.^57^, ^58^, ^59^ These imbalanced cytokines indirectly suppress haematopoiesis.

**FIGURE 1:**
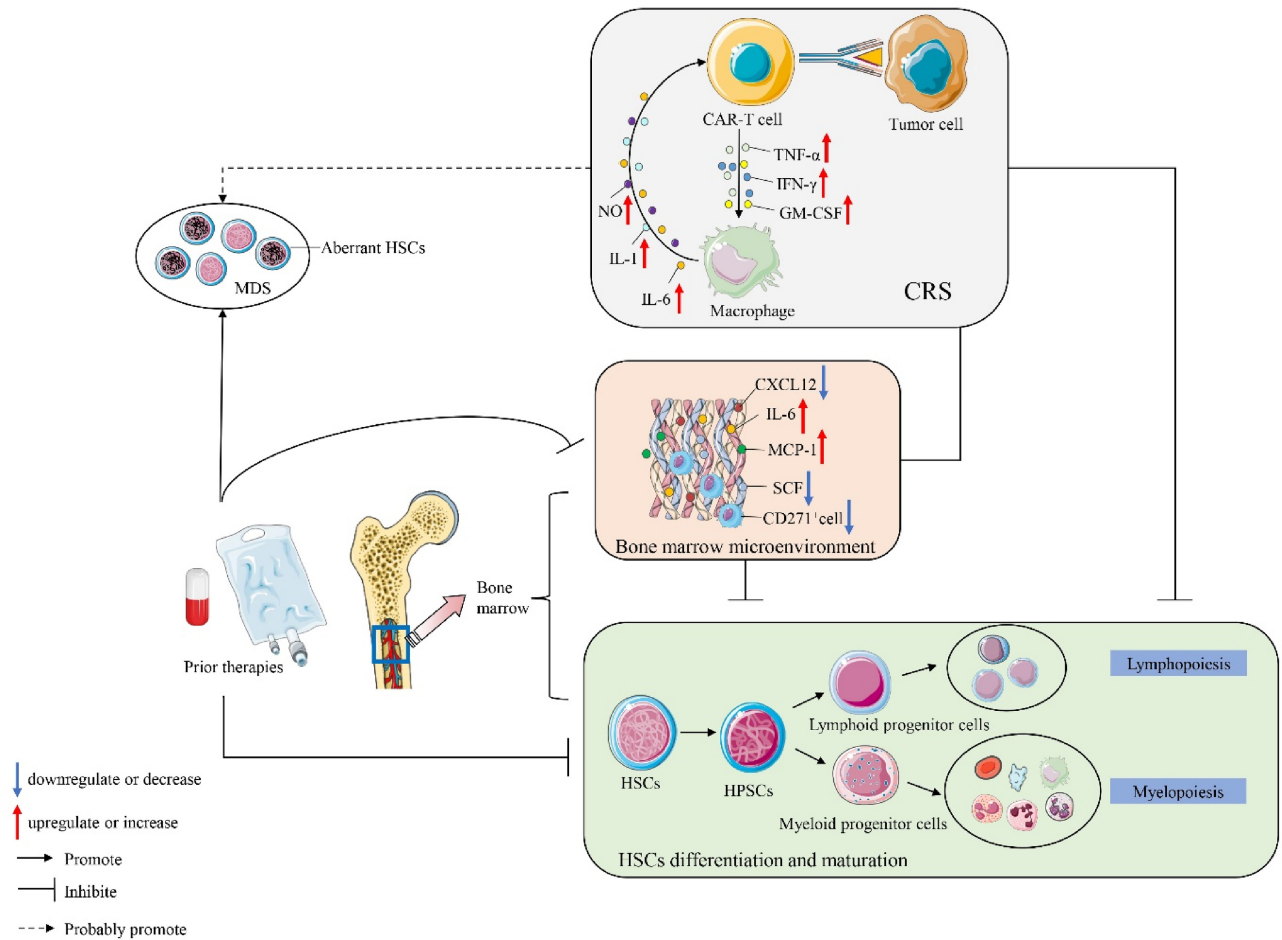
Potential mechanism of prolonged haematologic toxicity after CAR‐T cell therapy. CXCL12, C‐X‐C motif chemokine ligand 12; HPSCs, human pluripotent stem cells; HSCs, haematopoietic stem cells; IFN‐γ, interferon‐γ; IL, interleukin; MCP‐1, monocyte chemoattractant protein‐1; NO, nitric oxide; SCF, stem cell factor; TNF‐α, tumour necrosis factor‐alpha.

Figure 1 illustrates several potential mechanisms of PHT after CAR‐T‐cell therapy. First, the BM microenvironment is impaired, with decreased secretion of haematopoietic support factors and increased secretion of proinflammatory factors. Second, cytokines secreted by CAR‐T cells have direct or indirect impacts on HSCs self‐renewal and quiescence. Third, imbalanced cytokines might contribute to superior differentiation of a single lineage. Fourth, high‐strength chemotherapy prior to CAR‐T‐cell infusion can directly impair the proliferative capacity of HSCs or lead to clonal haematopoiesis, with insufficient baseline haematopoietic function, hypocellular BM or MDS after infusion.

## 5 CONSIDERATION FOR PROPHYLAXIS OF PROLONGED HAEMATOLOGIC TOXICITY

As mentioned above, baseline characteristics cannot be corrected. In light of the high grade of CRS associated with prolonged cytopenia, some studies have focused on reducing the severity of CRS and have shown that prophylactic or early use of prednisone and tocilizumab might be a promising strategy to prevent the occurrence of high‐grade CRS while not affecting CAR‐T‐cell efficacy.^60^, ^61^, ^62^, ^63^ Unfortunately, these studies have not reported information regarding the incidence of PHT.

## 6 CONSIDERATION FOR TREATMENT OF PROLONGED HAEMATOLOGIC TOXICITY

### 6.1 Granulocyte colony‐stimulating factor

In addition to symptomatic supportive treatment, such as red blood cell transfusion and platelet transfusion, granulocyte colony‐stimulating factor (G‐CSF) is the main and common therapeutic method for severe cytopenia. Society for Immunotherapy of Cancer clinical practice panels recommend that G‐CSF should be administered for persistent neutropenia (ANC <0.5 × 10^9^/L) on Day 28 after CAR‐T‐cell infusion and not proposed for use until there is no longer a risk of CRS (usually 2 weeks).^64^

### 6.2 Prednisone

Previous studies have demonstrated that prednisone has a bidirectional regulatory effect on the immune system. On the contrary, prednisone can inhibit the proinflammatory cytokine production induced by epithelial cells, dendritic cells and macrophages and cytotoxic immune responses by increasing IFN‐γ expression. On the contrary, prednisone can enhance T‐cell activation, which leads to active efficient T cells, generation of follicular helper T cells and production of antibodies by B cells.^65^ Moreover, prednisone can reduce apoptosis in erythroid progenitor cells and promote their survival in Diamond–Blackfan anaemia.^66^ In terms of the role of prednisone in stimulating BM haematopoiesis, it was used as treatment for PHT in the Wang cohort. Seventeen patients who developed PHT and for whom erythropoietin, platelet receptor agonists, transfusion, or G‐CSF previously failed received low‐dose prednisone (at a dosage of 0.5 mg/kg/day); all patients achieved haematologic recovery with a median response time of 21 (range 7–40) days. In addition, the side effects induced by prednisone, such as hypertension and hyperglycaemia, were alleviated with symptomatic treatment.^14^ Although there is no definitive evidence for the mechanism by which prednisone alleviates PHT after CAR‐T‐cell therapy, small doses are safe and effective for PHT.

### 6.3 Sirolimus

Sirolimus is an immunosuppressant that inhibits antigen‐induced T‐ and B‐cell proliferation and antibody production. Previous studies have verified that sirolimus is a promising treatment choice for patients with autoimmune hemocytopenia, such as AA, immune thrombocytopenia (ITP) and pure red cell AA.^67^, ^68^, ^69^ One case report described a patient who experienced prolonged severe cytopenia with active bleeding after CAR‐T‐cell therapy, with no response to dexamethasone, intravenous immunoglobulin (IVIG), recombinant human thrombopoietin (rhTPO), eltrombopag or avatrombopag, and eventually achieved complete haematopoietic recovery after administration of sirolimus.^70^ Given the single case, more clinical studies are needed to confirm the effectiveness and safety of sirolimus in treatment of PHT after CAR‐T‐cell therapy.

### 6.4 Thrombopoietin receptor agonists

Thrombopoietin receptor agonists (TPO‐RAs) are used for treatment of ITP, AA, chemotherapy‐induced thrombocytopenia and thrombocytopenia post stem cell transplantation.^71^, ^72^, ^73^, ^74^ TPO‐RAs bind to MPL (also known as TPO receptor), leading to its conformational change and activating a wide range of downstream signalling pathways, such as JAK2/STAT3/5, ERK1/2 and PI3K/Akt, and ultimately increasing megakaryocyte differentiation and maturation and platelet production.^75^, ^76^ Furthermore, as the TPO receptor is expressed on stem cells, these agents have a critical role in stem cell proliferation and maintenance.^77^ Eltrombopag, romiplostim and rhTPO are all TPO‐RAs and bind to MPL, activating similar signalling pathways. However, they differ in action region, degradation metabolic pathways, duration of action and metal chelation mechanisms, which may lead to synergistic effects when used together. Du et al. reported one patient who developed isolated thrombocytopenia at 15 months after CAR‐T‐cell infusion, with a high TPO concentration level and platelet autoantibody positivity. This patient had no response to platelet infusions, IVIG or IL‐11, but the combination therapy of rhTPO and eltrombopag was effective.^78^ A case series of four patients who developed PHT in the Nagle cohort received eltrombopag at daily dosages from 50 to 150 mg; surprisingly, all four patients achieved haematologic recovery, with a median of 123 (range 41–145) days.^27^ Moreover, six patients with prolonged cytopenia in the Beyar‐Katz cohort were administered TPO‐RAs (eltrombopag, *n* = 4; romiplastim, *n* = 1, both drugs, *n* = 1), which resulted in sustained haematologic responses.^79^ Drillet et al. reported that 10 of 11 patients with prolonged thrombocytopenia treated with TPO‐RAs achieved platelet recovery (PLT >50 × 10^9^/L) after a median time of 46 days.^80^ Notably, all PHT patients in the Sarah and Beyar‐Katz cohorts showed hypocellular or aplastic marrow prior to TPO‐RAs treatment, indicating at least partial overlap with AA with regard to pathogenesis. However, more clinical studies are needed to explore the mechanism and efficacy of TPO‐RAs for PHT after CAR‐T‐cell therapy.

### 6.5 Haematopoietic stem cell boost

Haematopoietic stem cell boost (HSCB), either as consolidation treatment or to treat prolonged cytopenia, has been performed in patients who had a complete response or complete response with incomplete haematologic recovery. HSCBs are categorized according to the source of stem cells into autologous (auto) and transplant donor derived (allogeneic; allo). In two case reports, auto‐HSCB reversed CAR‐T‐cell‐mediated prolonged pancytopenia in lymphoma and multiple myeloma patients.^81^, ^82^ Similarly, allo‐HSCB improved the prognosis of two B‐ALL patients with prolonged cytopenia and infection following CAR‐T‐cell therapy.^83^, ^84^ In addition to case reports, a multicentre study reported results for 31 patients receiving HSCB (auto, *n* = 30; allo, *n* = 1) for sustained severe and moderate neutropenia. A total of 84% (26/31) of patients achieved neutrophil recovery or improvement.^85^ Another study reported that four of seven patients with PHT following CAR‐T‐cell therapy responded to allo‐HSCB, with two achieving complete haematologic recovery at the last follow‐up.^86^ All patients in the above studies developed prolonged cytopenia after receiving BM aspirate before HSCB and had a considerably hypocellular marrow. Although use of HSCBs has been reported in small series, it has been indicated that HSCBs are suitable for hypocellular marrow, highlighting the safety and efficacy of HSCBs in patients with severe prolonged cytopenia following CAR‐T‐cell therapy.

### 6.6 Hypomethylating agents

Given that the majority of patients with secondary MDS after CAR‐T‐cell therapy are not candidates for allo‐HSCT due to donor availability and individual status, hypomethylating agents (HMAs) are likely to become a potential treatment strategy. Klimek et al. reported that HMAs show comparable activity in therapy‐related MDS (38% overall response rate) as in de novo MDS.^87^ Two studies have reported HMAs application in secondary MDS after CAR‐T‐cell therapy. One study reported that one patient achieved transient haematologic improvement after three cycles of azacytidine; unfortunately, she died due to recurrent pancytopenia.^49^ In the other study, two patients received azacytidine and decitabine. Although the MDS outcome is not available, they maintained ongoing complete response for primary disease and were still alive at the last follow‐up.^43^ The efficacy of HMA in PHT after CAR‐T‐cell therapy needs to be further investigated.

## 7 OVERALL SURVIVAL BETWEEN NON‐PHT AND PHT SUBGROUPS IN MM PATIENTS WHO RECEIVE CAR‐T‐CELL THERAPY

**FIGURE 2:**
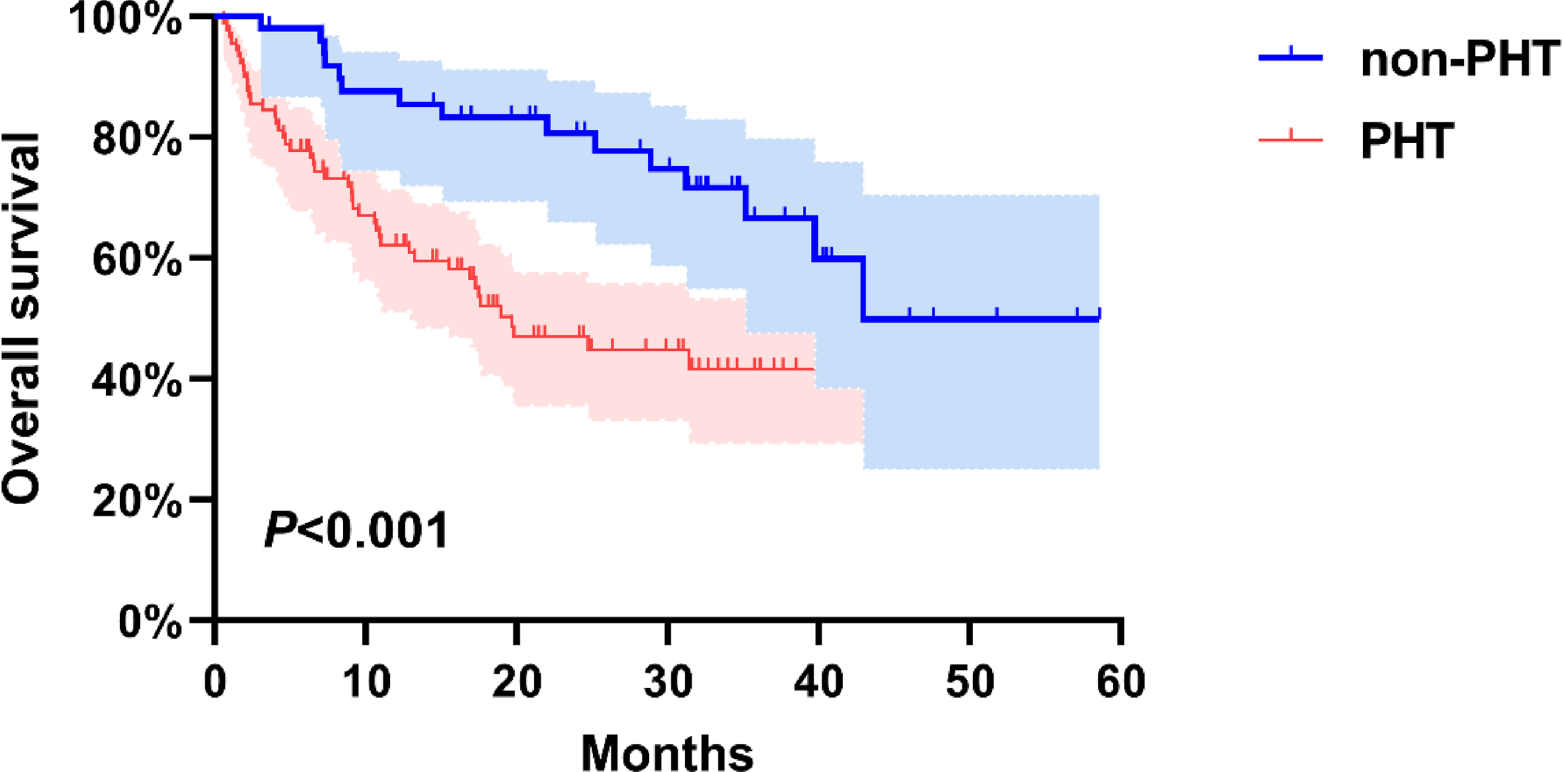
Overall survival were compared between MM patients with PHT and non‐PHT by Kaplan–Meier curves. Two‐sided *p* values were calculated based on the log‐rank test. PHT, prolonged haematological toxicity.

Owing to the limited reports on the overall survival (OS) of PHT and non‐PHT patients after CAR‐T‐cell therapy, only two studies with available data for R/R MM patients who received CAR‐T cells were obtained by Engauge Digitize software version 4.1. Analysis was performed using GraphPad Prism software version 9.0. The results showed that PHT patients had worse prognosis, with an estimated 3‐year OS of 66.5% compared to 41.5% in non‐PHT patients (*p* < 0.001) (Figure 2).

## 8 FUTURE PERSPECTIVES

With rapid commercialization of CAR‐T‐cell therapy, a growing number of clinical trials have reported PHT development. The majority of PHT cases seem to resolve by 3 months after CAR‐T‐cell infusion. Nevertheless, persistent and incorrigible cytopenia or late‐onset cytopenia has occurred in a small number of patients. The aetiology of prolonged cytopenia is unclear, and the various hypotheses and related factors proposed in various prediction models are not consistent, which makes it difficult to implement precise clinical intervention.

In general, the damaged BM microenvironment and HSCs, clonal haematopoiesis prior to CAR‐T‐cell therapy, and the influence of imbalanced cytokines on haematopoiesis are involved in the pathogenesis of PHT. Previous studies have demonstrated that PHT is associated with increased risk of infection, longer hospitalization and poor prognosis after CAR‐T‐cell infusion. Therefore, early clinical treatment plays a crucial role in management of PHT. As noted, BM biopsy is recommended to rule out secondary myeloid malignancies such as MDS, especially for those with persistent severe cytopenia lasting more than 1 month or extremely late‐onset cytopenia.

American Society of Clinical Oncology guidelines recommend that G‐CSF and prednisone may be used in cases with PHT not related to MDS, but there is no mention of the time or dosage of use. Although PHT is confronted with clinical challenges as a result of a lack of guidelines for management, attempts to use TPO‐RAs in PHT patients have achieved surprising efficacy. Nevertheless, long‐term efficacy and safety are unknown. Owing to the possibility of TPO‐RAs promoting clonal haematopoiesis, TPO‐RAs should be discontinued for PHT patients who do not respond after 3 months of treatment. In addition, reports on application of HSCB in treatment of PHT are gradually increasing. We propose that stem cells from a prior autologous or an allogeneic donor be persevered for those with a high risk of developing PHT, such that patients who achieve complete response will be accepted for HSCB for subsequent rescue in the event of prolonged cytopenia.

## 9 CONCLUSIONS

Ultimately, there are some weaknesses in this article. The majority of listed studies only reported the incidence of PHT and were inconsistent in the definition of endpoint duration and differed in recommended management, with a lack of comprehensive management guidelines. Therefore, prospective studies are required, and the relevant mechanism of PHT should be explored in clinical trials. Once the pathophysiology of PHT is better understood, precise treatment strategies may emerge. It is anticipated that potential late HT will be overcome in the future with exponential application of CAR‐T cells and a deepened understanding of PHT.

## AUTHOR CONTRIBUTIONS

**Qi Liu:** Writing – original draft (lead). **Tonglin Hu:** Writing – original draft (equal). **Hangchao Li:** Writing – original draft (supporting). **Yingying Shen:** Writing – original draft (supporting). **Dijiong Wu:** Writing – review and editing (equal). **Baodong Ye:** Writing – review and editing (equal).

## FUNDING INFORMATION

The present study was supported by the National Natural Science Foundation of China (No. 82274273, 82174138), Zhejiang Scientific Research Fund of Traditional Chinese Medicine (No. 2020ZB085, No. 2022ZA059), Health Technology Plan of Zhejiang Province (No. 2022RC216) and Project of Zhejiang Famous Traditional Chinese Medicine Expert Inheritance Studio Construction under Grant No. GZS2021022.

## CONFLICT OF INTEREST STATEMENT

The authors declare no competing interests.

## Supporting information


Table S1.

## Data Availability

The data used to support the study are available from the corresponding author upon request.
