# Supplementary material for: Prolonged haematologic toxicity in CAR‐T‐cell therapy: A review
Source: J Cell Mol Med. 2023 Sep 13;27(23):3662–71. doi: 10.1111/jcmm.17930 (PMC10718150; doi:10.1111/jcmm.17930)
Supplement: Supplementary file 1 — Table S1. [file JCMM-27-3662-s001.docx]

**Supplementary Table 1** Basic characteristics and incidence of PHT of the typical studies. PHT: prolonged hematological toxicity, R/R: relapsed or refractory, ALL: acute lymphoblastic leukemia, DLBCL: diffuse large B-cell lymphoma, MM: multiple myeloma, LBCL: large B-cell lymphoma, HL: hodgkin lymphoma, NHL: non-hodgkin lymphoma, ANC: absolute neutrophil count, PLT: platelet, B-cell maturation antigen (BCMA), axi-cel: axicabtagene ciloleucel, ide-cel: idecabtagene vicleucel, liso-cel: lisocabtagene maraleucel.

a: hematologic toxicity was analyzed in 29 responding patients

b: all the included achieved CR at Day 28

c: data were missing [neutropenia (n=3), anemia (n=2), thrombocytopenia (n=2)]

d: hematologic toxicity was analyzed in 19 patients with ongoing CR and no diagnosis of MDS

e: patients were no longer included in analysis if receiving subsequent treatment, second CAR-T cell infusion and withdraw from the study. A total of 157, 34, 8, 8 and 9 were included in hematologic toxicity analysis at day 28, 60, 90, 120 and 180, respectively

| Study (and DOI) | Trial sequence | Phase or study design | Disease | Sample | Target | Costimulatory domain | Definition of PHT | Incidence of PHT |
| --- | --- | --- | --- | --- | --- | --- | --- | --- |
| Fried 2019 | NCT#02772198 | 1b/2 | R/R B-cell ALL and NHL | 35^a^ | CD19 | CD28 | Neutropenia, anemia or thrombocytopenia on day 21, severe neutropenia was defined as ANC<0.5×10^9^/L, severe thrombocytopenia was defined as PLT<50×10^9^/L, severe anemia was defined as anemia required red blood cells infusion | 93% (27/29) of patients developed PHT while 38% (11/29) for severe PHT; 76% (22/29) for neutropenia and severe in 10 (34%), 76% (22/29) for thrombocytopenia and severe in 6 (21%), 17% (5/29) patients required RBC transfusion |
| Li 2022 | ChiCTR-OIC-17011272 | 1/2 | R/R MM | 54 | BCMA+CD19 | 4-1BB | Severe neutropenia (ANC<0.5×10^9^/L), severe anemia (HB<60 g/L), or severe thrombocytopenia (PLT<20×10^9^/L) on day 28 post-infusion | 52% (28/54) of patients developed PHT; 46% for severe neutropenia, 30% for severe anemia and 31% for severe thrombocytopenia |
| Wang 2023 | ChiCTR-ONN-16009862  ChiCTR1800015164 | retrospective study | R/R B-ALL | 109^b^ | CD19 | CD28 | Any grade neutropenia, anemia or thrombocytopenia on day 28 | 78.9% (86/109) of patients developed PHT |
| Schuster 2019 | NCT02445248 | 2a | R/R DLBCL | 111 | CD19 | 4-1BB | Unresolved prolonged grade 3 or 4 thrombocytopenia or neutropenia at day 28 | 32% of patients developed PHT; 24% for neutropenia, 41% for thrombocytopenia |
| Schuster 2021 | NCT02445248 | 2 | R/R LBCL | 115 | CD19 | 4-1BB | Grade 3 or worse cytopenia not resolved by day 28 | 34% (39/115) of patients developed PHT |
| Goto 2020 | NCT02445248 | 2 | R/R DLBCL | 9 | CD19 | 4-1BB | Grade 3/4 cytopenia not resolved by day 28 | 77.8% (7/9) of patients developed PHT |
| Ghorashian 2019 | NCT02443831 | 1 | R/R and pediatric ALL | 14 | CD19 | 4-1BB | Grade 3/4 cytopenia persisting beyond day 28 or recurring after this time point | 71.4% (10/14) of patients developed PHT; 43% (6/14) for neutropenia and 21.4% (3/14) for thrombocytopenia |
| Maude 2018 | NCT02435849 | 1/2a | Children and young adults with R/R B-ALL | 75 | CD19 | 4-1BB | Grade 3/4 neutropenia or thrombocytopenia not resolved by day 28 | 53% (40/75) of patients developed prolonged neutropenia and 41% (31/75) of patients developed prolonged thrombocytopenia |
| Sesques 2020  Data were missing [Neutropenia (n=3)  Anemia (n=2)  Thrombocytopenia (n=2)] | Commercial  CAR T-cell | Retrospective study | R/R aggressive B-cell lymphoma | 61^c^ | CD19 | CD20+4-1BB | Grade 3/4 neutropenia, anemia or thrombocytopenia not resolved by day 28 | 36% (21/58) of patients developed prolonged neutropenia, 14% (8/59) of patients developed prolonged anemia and 48% (28/59) of patients developed prolonged thrombocytopenia |
| Sehgal 2022 | NCT03483103 | 2 | R/R LBCL | 61 | CD19 | 4-1BB | Grade 3 or worse neutropenia, anemia or thrombocytopenia not resolved on day 28 | 30% (18/61) of patients developed PHT |
| Zhou 2020 | ChiCTR-  OOC-16007779 | 1 | R/R B-cell NHL | 21 | CD19 | Forth generation (CD28+CD27) | Grade 3/4 of cytopenia for more than 28 days or 3 months | 29% and 14% of patients developed prolonged neutropenia more than 28 days and 3 month, 14% and 0 of patients developed prolonged anemia more than 28 days and 3 month, 29% and 10% of patients developed thrombocytopenia more than 28 days and 3 month, respectively |
| Wang 2021 | ChiCTR-ORN-16008948  ChiCTR1800017402  ChiCTR1800015575 | Retrospective study | R/R ALL | 76 | CD19,CD22, CD19/CD22 | 4-1BB | Severe neutropenia (ANC<0.5×10^9^/L), severe anemia (HB<60 g/L), or severe thrombocytopenia (PLT<20×10^9^/L) on day 28 post-infusion | 25% (19/76) of patients developed PHT |
| Kitamura 2023 | Commercial  CAR T-cell | Retrospective study | R/R DLBCL | 21 | CD19 | CD28+4-1BB | Cytopenia after 3 to 4 weeks | 66.7% (14/21) of patients developed PHT |
| Makita 2022 | NCT03484702 | 2 | R/R LBCL | 10 | CD19 | 4-1BB | Grade of 3 or worse of neutropenia, anemia or thrombocytopenia not resolved by day 29 | 60% (6/10) of patients developed PHT |
| Abramson 2020 | NCT02631044 | 1 | R/R LBCL | 269 | CD19 | 4-1BB | Grade ≥ 3 neutropenia, anemia or thrombocytopenia not resolved at day 29 | 37% (100/269) of patients developed PHT; 16% (43/269) for neutropenia, 4% (11/269) for anemia and 22% (58/269) for thrombocytopenia |
| Wang 2022 | ChiCTR1800017404 | Retrospective study | R/R MM | 93 | BCMA | 4-1BB | Grade 3 or 4 neutropenia, anemia or thrombocytopenia after day+30 | 72.04% (67/93) of patients developed PHT; 38.71% (36/93) for neutropenia, 22.58% (21/93) for anemia and 59.14% (55/93) for thrombocytopenia |
| Sarah 2021 | Commercial  CAR T-cell | Retrospective study | R/R DLBCL | 31 | CD19 | CD28+4-1BB | Grade 3 or 4 neutropenia or thrombocytopenia after day+30 | 58% (18/31) of patients developed PHT; All 18 patients with PHT had grade 3 or 4 neutropenia while 83% (15/18) patients had grade 3 or 4 thrombocytopenia |
| Strati 2021 | NCT02348216  NCT03153462 | ZUMA-1+ZUMA-9 | R/R LBCL | 31 | CD19 | CD28 | Grade 3/4 of neutropenia, anemia or thrombocytopenia at day 30 | 48% (15/31) of patients developed PHT; 29% (9/31) for neutropenia, 16% (5/31) for anemia and 42% (13/42) for thrombocytopenia |
| Jacobson 2022 | NCT03105336 | 2 | R/R NHL | 148 | CD19 | CD28 | Grade 3 or worse cytopenia present on or after day 30 | 34% (50/148) of patients developed PHT |
| Kato 2022 | JapicCTI-183914 | 2 | R/R LBCL | 16 | CD19 | CD28 | Any cytopenia with duration ≥ 30 days or any consecutive events of cytopenia with combined duration ≥ 30 days | 18.8% (3/16) of patients developed prolonged neutropenia, 6.3% (1/16) of patients developed prolonged anemia and 6.3% (1/16) of patients developed prolonged thrombocytopenia |
| Neelapu 2022 | NCT03761056 | 2 | High-risk LBCL | 40 | CD19 | CD28 | Grade ≥ 3 cytopenia present on or after day 30 | 20% (8/40) of patients developed PHT |
| Shah 2021 | NCT02614066 | 2 | R/R B-cell ALL | 55 | CD19 | CD28 | Grade 3 or worse cytopenia were present on or after day 30 | 36% (20/55) of patients developed PHT; 25% (14/55) for neutropenia, 7% (4/55) for anemia and 18% (10/55) for thrombocytopenia |
| Topp 2021 | NCT02348216 | 1/2 | R/R LBCL | 41 | CD19 | CD28 | Grade ≥ 3 neutropenia, anemia or thrombocytopenia present on or after day 30 | 39% (16/41) of patients developed PHT; 32% (13/41) for neutropenia, 7% (3/41) for anemia and 10% (4/41) for thrombocytopenia |
| Pan 2021 | ChiCTR2000034762 | 1 | R/R T-ALL | 20  New donor-derived CAR-T cells (n=8), previous SCT donor-derived CAR-T cells (n=12) | CD7  (donor-derived) | 4-1BB | Grade 3/4 neutropenia, anemia or thrombocytopenia 1 month after infusion | 71% (5/7) and 83% (10/12) of patients developed PHT in new and previous SCT donor-derived CAR-T cells group, respectively |
| Munshi 2021 | NCT03361748 | 2 | R/R MM | 128 | BCMA | 4-1BB | Grade 3/4 neutropenia or thrombocytopenia 1 month after infusion | 41% (52/128) for neutropenia and 48% (62/128) for thrombocytopenia |
| Rodriguez-Otero 2023 | NCT03651128 | 3 | R/R MM | 386  Ide-cel (n=254, 225 received an ide-cel infusion), standard regimen (n=132) | BCMA | 4-1BB | Grade 3 or 4 neutropenia or thrombocytopenia not resolved before 1 month | 40% (89/225) of patients developed prolonged neutropenia and 37% (84/225) of patients developed prolonged thrombocytopenia |
| Kamdar 2022 | NCT03575351 | 3 | R/R LBCL | 184  liso-cel (n=92), standard of care (n-92) | CD19 | 4-1BB | Grade 3 or worse cytopenia at 35 days after liso-cel infusion or after the start of the last chemotherapy in the standard of care cohort | 43% (40/92) and 3% (3/92) of patients developed PHT in liso-cel and standard of care group, respectively |
| Benjamin 2020 | NCT02746952 | 1 | R/R B-cell ALL | 25 | CD19 | UCART19  allogeneic | Grade 4 of neutropenia or thrombocytopenia persisting beyond day 42 | 16% (4/25) of patients developed PHT |
| Nahas 2019 | Commercial axi-cel | Retrospective study | Refractory aggressive B cell lymphomas | 21 | CD19 | CD28 | ANC<0.5×10^9^/L or requiring filgastrim to maintain ANC>0.5×10^9^/L after day 42 | 38% (8/21) of patients developed PHT |
| Cordeiro 2019 | NCT01865617 | 1/2 | R/R ALL, NHL, or CLL | 86^d^ | CD19 | 4-1BB | Requiring RBC or platelet transfusion or growth factor support beyond 90 days after CAR-T cell infusion | 16% (3/19) of patients developed PHT; 10.5% (2/19) for pancytopenia requiring RBC and PLT transfusion, 5.3% (1/19) for severe neutropenia receiving G-CSF |
| Wang 2020 | NCT02601313 | 2 | R/R MCL | 68 | CD19 | 4-1BB | Grade 3 or higher neutropenia, anemia or thrombocytopenia more than 90 days | 26% of patients developed PHT; 16% for neutropenia, 12% for anemia and 16% for thrombocytopenia |
| Locke 2019 | NCT02348216 | 1/2 | R/R LBCL | 108 | CD19 | CD28 | Grade 3 or worse cytopenia at 3 month or later | 17% (18/108) of patients developed PHT; 11% (12/108) for neutropenia, 7% (8/108) for thrombocytopenia and 3% (3/108) for anemia |
| Ramos 2020 | NCT02690545 NCT02917083 | 1/2 | R/R HL | 42 | CD30 | CD28 | Grade 3/4 neutropenia or thrombocytopenia not resolved by month 3 | 10% (4/42) of patients developed PHT; 10% (4/42) for thrombocytopenia, none for neutropenia |
| Juluri 2022 | NCT01865617 | 1/2  Retrospective study | R/R B-cell malignancies (ALL, NHL, CLL) | 173^e^ | CD19 | CD28+4-1BB | Grade 3 or worse neutropenia, anemia or thrombocytopenia at day 28, 60, 90, 120, 180 | 45.9% (72/157), 29.4% (10/34), 37.5% (3/8), 25% (3/8), 33.3% (3/9) of patients developed neutropenia at day 28, 60, 90, 120, 180; 14.6% (23/157), 20.6% (7/34), 25% (2/8), 37.5% (3/8), 11.1% (1/9) of patients developed anemia at day 28, 60, 90, 120, 180; 33.8% (53/157), 26.4% (9/34), 50% (4/8), 50% (4/8), 33.3% (3/9) of patients developed thrombocytopenia at day 28, 60, 90, 120, 180 |
